# Seroprevalence of severe fever with thrombocytopenia syndrome virus in China: A systematic review and meta-analysis

**DOI:** 10.1371/journal.pone.0175592

**Published:** 2017-04-11

**Authors:** Peng Li, Zhen-Dong Tong, Ke-Feng Li, An Tang, Ya-Xin Dai, Jian-Bo Yan

**Affiliations:** 1 Zhoushan Municipal Center for Disease Control and Prevention, Zhoushan, Zhejiang, P.R. China; 2 Zhejiang Provincial Key Laboratory of Health Risk Factors for Seafood, Zhoushan Municipal Center for Disease Control and Prevention, Zhoushan, Zhejiang, P.R. China; University of Minnesota, UNITED STATES

## Abstract

**Objective:**

Severe fever with thrombocytopenia syndrome (SFTS) is an emerging infectious disease caused by a novel bunyavirus-SFTSV. The seroprevalence of anti-SFTSV antibodies including immunoglobulin G (IgG) and immunoglobulin M (IgM), specific to SFTSV in the general population has been investigated in various epidemiological studies with inconsistent results. Here, we clarify this discrepancy and reach a more comprehensive result by mean of a meta-analysis.

**Methods:**

All relevant articles were searched in the electronic databases (PubMed, Web of science, Embase, Chinese National Knowledge Infrastructure database, Chinese Wanfang database) up to November 2016. The pooled seroprevalence and 95% confidence intervals (95% CIs) were calculated by random- or fixed- model on the basis of heterogeneity.

**Results:**

In total, 21 studies containing 23,848 blood samples from 7 provinces were included in this meta-analysis. The minimum and maximum reported seroprevalences of SFTSV among humans in China were 0.23% and 9.17%, respectively. The overall pooled seroprevalence of SFTSV antibodies was 4.3% (95%CI: 3.2%-5.5%). The pooled prevalence was 5.9% (95%CI: 4.7%-7.0%) in Zhejiang province, 4.9% (95%CI: 4.1–5.8%) in Anhui province, 3.9% (95%CI: 1.3%-6.4%) in Shandong province, and 0.7% (95%CI: 0.2%-1.1%) in Jiangsu province. Stratified by occupation, the pooled prevalence of farmer was 6.1% (95%CI: 3.4%-8.9%) and others (mainly are students) was 3.3% (95%CI: 2.4%-4.2%). Additionally, seroprevalence of SFTSV in people who lived in the same village with the patient were higher than that of people who lived in a different village. Seropositive rates in sampling years after 2012 were higher than that before 2012. The prevalence of SFTSV did not differ by age or gender. Sensitive analysis by omitting one study at a time indicated the results of the pooled seroprevalence were robust.

**Conclusions:**

Seroprevalence of SFTSV among healthy population in central and eastern China is high. Surveillance efforts on mild or asymptomatic infections among endemic persons are needed.

## Introduction

Severe fever with thrombocytopenia syndrome (SFTS) is a notifiable infectious disease characterized by fever, weakness, leukopenia, thrombocytopenia, gastrointestinal symptoms, and central nervous system manifestations [1–3]. The causative agent of SFTS is a novel member of the Phlebovirus in the family Bunyaviridae, SFTS virus (SFTSV), which was first isolated from human beings in rural areas of central China by Yu et al in 2009 [1]. About the latter, the disease was also reported in Korea and Japan in 2012, and a disease similar to SFTS has been reported in the United States [4, 5]. More recently, human cases have been widely covered in at least 16 provinces in east and center of China, including Shandong, Zhejiang, Jiangsu, Anhui, Henan, Hubei, and Liaoning provinces, etc [6]. The incidence of the disease is very high in some epidemic areas with a case-fatality rate of up to 30%, which has posed an increasingly threat to global health [2].

Time range from March to November is the epidemic season of SFTS, May-July is the peak time of SFTSV infection. SFTSV is most likely to be transmitted by tick bite according to evidence from tick exposure history and SFTSV detection in ticks such as Haemaphysalis longicornis ticks, and virus gene sequence analysis showed the virus in ticks closely related to those circulating in humans [7–10]. High seroprevalence to SFTSV has been reported in domestic animals such as goats, sheep, cattle, dogs, etc and small mammals such as rodent and shrews. However, the host range of the virus and the role of these animals in the transmission of SFTSV is poorly understood. [11, 12]. In addition, person to person transmission by contacting with infected patient’s blood or mucous has been reported in China [13–15].

In past years, seroprevalence of SFTSV among healthy population has been widely investigated in various epidemiologic studies. Current estimates of the seroprevalence of SFTSV among general humans in China are almost based on one or several villages with relatively small sample size in one province rather than nationally representative sample of this population. In addition, seroprevalence of SFTSV among healthy humans in different gender, age groups, and endemic regions has yet to be illuminated. Meta-analysis is an effective method of pooling data of individual study together, thus enhancing the statistical power of the analysis for the estimates, to reach a more comprehensive result. It has been widely utilized in sero-epidemiological studies of infectious diseases, such as *Influenza A (H9N2)*, *Ebola and Marburg viruses*, and *Enterovirus 71*, etc [16–18]. In present study, we determine the overall pooled seroprevalence of SFTSV among healthy individuals in China by mean of a meta-analysis. We also estimate the pooled seroprevalence of total antibodies to SFTSV in different subgroups of healthy humans.

## Materials and methods

### Search strategy

All relevant articles about the seroprevalence of SFTSV in general population were searched via the following electronic databases: Pubmed, Web of Science, Embase, Chinese National Knowledge Infrastructure (CNKI), Chinese WanFang Database. Date searches were carried out up to November 2016, without restrictions regarding language, publication year and district. The following search words and terms in English and Chinese were used in the final search: (“seroprevalence” or “prevalence” or “serum” or “antibodies” or “seroepidemiology”) and (“SFTS” or “SFTSV” or “Severe fever with thrombocytopenia syndrome” or “Severe fever with thrombocytopenia syndrome virus”). Moreover, all relevant references cited in original articles and reviews were also manually searched to identify additional articles not indexed by these databases.

### Eligibility criteria

To obtain the valid articles we needed, the following criteria were established: 1) the study either reported the seroprevalence of SFTSV or had sufficient data for calculating the seroprevalence; 2) individuals neither had SFTS infection in the past, being hospitalized for any clinically similar disease, or contact with a person who had SFTS as defined previously [1]; 3) specific-SFTSV antibody or total antibodies (IgG and IgM) to SFTSV were tested by using a double-antigen sandwich enzyme-linked immune sorbent assay (ELISA) kit or indirect-ELISA. Exclusion criteria included abstracts, conferences, case reports, letters, duplicated publications and studies reporting on SFTSV among population with non-Chinese.

### Data extraction

After initial evaluation, two authors independently and carefully screened the articles on title and abstract according to the eligibility criteria. Authors also filled out a standard quality assessment checklist with 11 items concerning the methodological aspects of cross-sectional studies for each study [19]. If the studies were based on the same sample, only the study with greatest epidemiological quality was selected. The following information was extracted from every eligible article: first author, publication year, researched province, sampling time, sampling method, sample size, positive rate in each study, number of research spot and antibodies testing method. We also extracted the number of positive for SFTSV antibodies according to gender, age, occupation and province, in order to estimate the seroprevalence of SFTSV in subgroups. Data with discrepancies in identification were resolved through discussing, if there is no agreement, the third investigator would make an ultimate decision.

### Quality assessment

The quality of the included studies was assessed by Agency for Healthcare Research and Quality (AHRQ), which is consisted of 11-item with a yes/no/unclear response option: “No” or “unclear” was scored “0” and the “Yes” would be scored “1”[19]. Articles scored as 0–3, 4–7, 8–11 indicated low, moderate and high quality, respectively.

### Statistical analysis

According to the results of heterogeneity test in different groups, the proper model was adopted to assess a pooled value and 95%CI of seroprevalence of SFTSV antibodies in healthy population.

Statistical heterogeneity across different studies was assessed by using the Cochran Q and I^2^ statistics [20]. *P*-values of the Cochran Q test less than 0.1 is considered to be statistically significant, and I^2^ value more than 75% indicates high heterogeneity. A significant Q statistic (*P*-values<0.10) indicate heterogeneity across studies, and then the DerSimonian and Laird method in random effect model result is used for meta-analysis. When the *P*-value of heterogeneity test is more than 0.1 (*P*-values>0.10), the results of Mante-Haenszel method in a fixed-effect model will be adopt. In order to explore the potential source of heterogeneity between studies and the seroprevalences of SFTSV antibodies with different characters such as gender, age, occupation and province, subgroups analysis were also conducted. Additionally, a leave-one-out sensitivity analysis was carried out to assess the impact of each study on the overall pooled estimate.

The potential publication bias was assessed by using Begg’s funnel plot [21]. Funnel plot asymmetry was further assessed by the method of Egger’s linear regression test. If the *P*-value of Egger’s test was less than 0.05, statistically significant publication bias might exist.

All statistical analysis were performed using STATA 11.0 (Stata Corporation, College Station, Texas, USA).

## Results

### Search results

**Fig 1:**
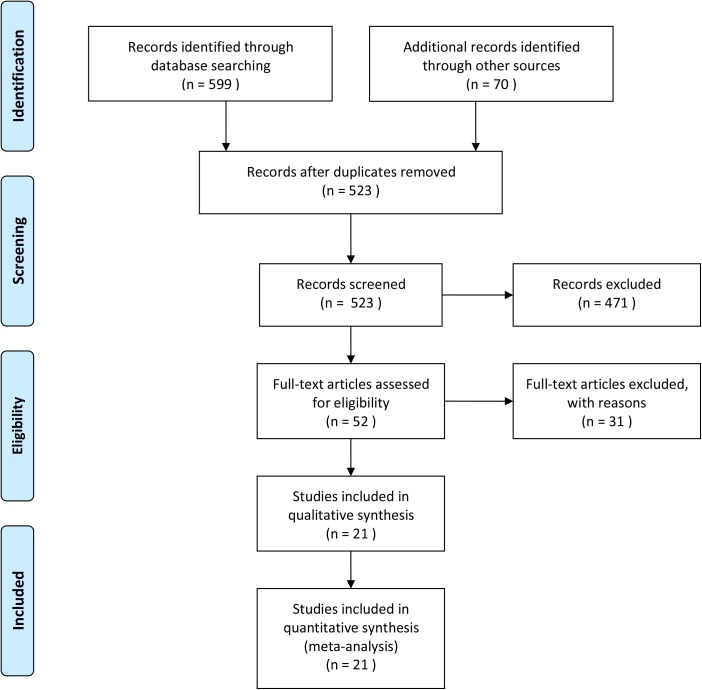
Flow chart of studies selection process in this meta-analysis.

The detailed study retrieval steps according to the PRISMA statement was shown in Fig 1. Initially, the search retrieved 669 relevant articles from the Pubmed, Web of Science, Embase, Chinese National Knowledge Infrastructure (CNKI), Chinese WanFang Database. Based on the eligibility criteria, we firstly removed 146 articles due to duplicate publications, and an additional 471 articles were excluded after review of the title and abstract for irrelevant topics or because they were abstracts/letters/reviews/comments/case reports. After carefully reviewing the full text and data of the remaining 52 articles, 31 ineligible records were excluded due to overlapping data or lack of some indicators. Finally, 21 studies containing 23, 848 blood samples were included in the final analysis. The basic characteristics and data extraction from these included studies was shown in **Table 1**.

**Table 1:** Basic characteristics of the included studies in this meta-analysis.

First author (ref.)	Publication year	Province	Sampling time	Sampling method	Region	Sample size	No. of positive	P* (%)	No. of research spot	Quality Score	Endemic	Language	Test method
Wei [22]	2015	SX	2014	NA	Rural	363	20	5.51	1 village	6	Y	ENG	D-ELISA
Hu [23]	2015	HN	2011.7–2013.12	Physical examination	Urban & rural	5245	343	6.54	213 towns	7	Mixed	ENG	ELISA
Zhan [24]	2013	HB	2010–2012	NA	Urban & rural	957	61	6.37	9 towns	6	Mixed	CHN	ELISA
Xing [25]	2015	HB	2012.8–2013.5	NA	Rural	419	35	8.35	7 villages	6	Y	ENG	D-ELISA
Wang [26]	2016	ZJ	2015.10–12	Randomly	Rural	200	15	7.50	2 villages	6	Mixed	CHN	ELISA
Zhang [27]	2014	ZJ	2013	NA	Rural	120	11	9.17	3 villages	6	Y	ENG	D-ELISA
Sun [28]	2015	ZJ	2013	Randomly	Rural	1380	76	5.51	6 districts	8	Y	ENG	ELISA
Tan [29]	2015	JS	2010~2011	NA	Rural	866	2	0.23	5 towns	6	Mixed	CHN	D-ELISA
Liang [30]	2014	JS	2011	NA	Urban & rural	2510	10	0.44	7 counties	7	Mixed	ENG	D-ELISA
Li [31]	2014	JS	2012.3–2013.1	Randomly	Rural	2547	33	1.30	6 counties	8	Y	ENG	D-ELISA
Zhang [32]	2011	JS	2010.7–11	NA	Rural	1922	17	0.94	6 districts	6	Y	CHN	D-ELISA
Jiao [33]	2011	JS&AH	2010	NA	Rural	250	9	3.60	2 districts	5	Y	ENG	D-ELISA
Huang [34]	2016	AH	2012.6	Randomly	Rural	270	17	6.30	8 towns	8	N	ENG	ELISA
Xu [35]	2015	AH	2013.9–10	Randomly	Rural	166	14	8.43	8 villages	6	Mixed	CHN	ELISA
Lyu [36]	2016	AH	2014–2015	NA	Rural	2126	99	4.66	12 villages	7	Y	ENG	ELISA
Niu [37]	2013	SD	2011	NA	Rural	2590	140	5.41	30 villages	8	Y	CHN	I-ELISA
Wang [38]	2013	SD	2010–2011	NA	Rural	315	4	1.27	1 county	6	Y	CHN	D-ELISA
Zhou [39]	2014	SD	2011. 4–12	Randomly	Rural	1525	138	9.05	NA	6	Y	CHN	I-ELISA
Luo [40]	2016	SD	2015.11–2016.1	Randomly	Rural	628	33	5.25	9 villages	8	Y	CHN	ELISA
Zhao [41]	2012	SD	2011.6	Convenience	Rural	237	2	0.84	2 villages	7	Y	ENG	D-ELISA
Cui [42]	2013	SD	2011	NA	Rural	78	1	1.30	5 villages	6	Y	ENG	D-ELISA

*Abbreviations*: SX, Shaanxi province; ZJ, Zhejiang province; JS, Jiangsu province; SD, Shandong province; AH, Anhui province; HN, Henan province; HB, Hubei province; ENG, English; CHN, Chinese; NA, not available.

P*(%), positive rate for SFTSV-specific IgG or IgM by using a double-antigen sandwich enzyme-linked immunosorbent assay (D-ELISA) or indirect-ELISA.

### Characteristics of included studies

**Fig 2:**
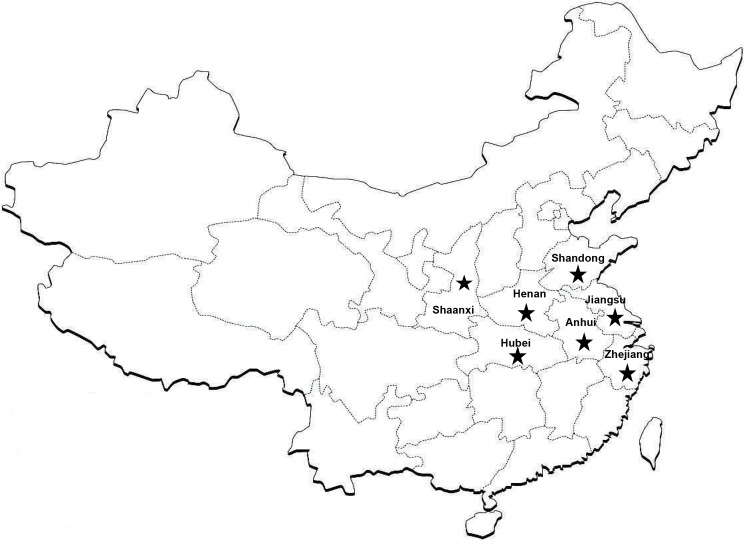
Location of referred provinces in China where serum samples of healthy persons were collected and tested for the presence of severe fever with thrombocytopenia syndrome virus–specific IgG and IgM.

For these 21 articles, sample sizes across the studies ranged from 78 to 2590. 12 of the studies were published in English and 9 in Chinese. 6 studies were of high quality, other studies were of moderate quality (**Table 1**). Generally, the most prevalence studies were conducted in the center and east of China (Shaanxi [22], Henan [23], Hubei [24, 25], Zhejiang [26–28], Jiangsu [29–32], Jiangsu&Anhui [33], Anhui [34–36], and Shandong provinces [37–42]), the location of referred provinces in China was shown in **Fig 2**.

### Overall seroprevalence of SFTSV

**Fig 3:**
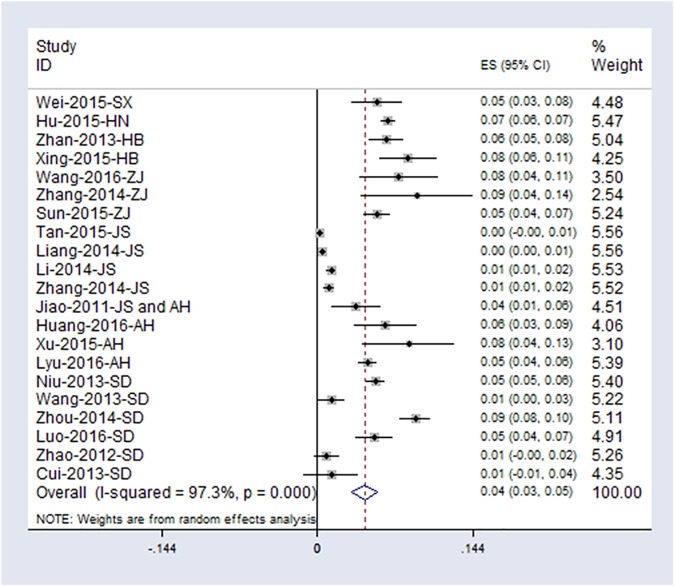
Forest plot of seroprevalence for total antibodies against SFTSV in healthy population. The middle point of each line indicates the prevalence rate and the length of line indicates 95% confidence interval of each study. Rhombus shape indicates 95% confidence interval for all studies.

Seroprevalence of SFTSV antibodies varied from 0.23% to 9.17% and were displayed as forest plots in **Fig 3**. The heterogeneity across the studies was high (Q = 774.93, *P*<0.001; *I*^*2*^ = 97.3%). The overall pooled seroprevalence estimate for SFTSV in random effect model was 4.3% (95%CI: 3.2%-5.5%).

### Subgroup analysis

**Fig 4:**
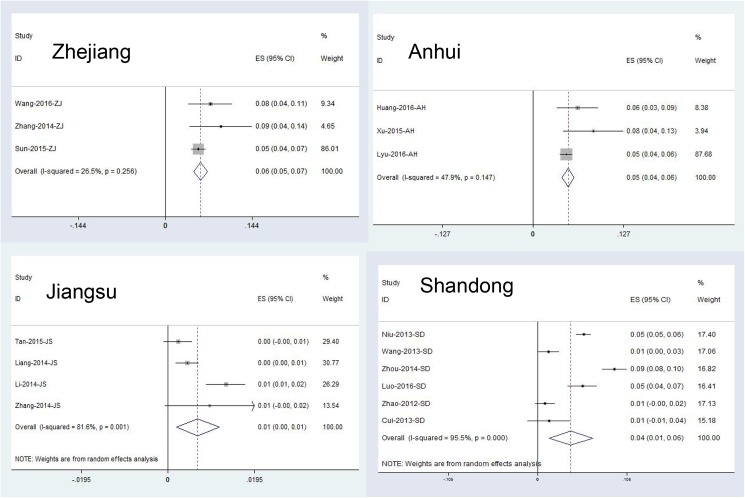
Forest plots of seroprevalence for total antibodies against SFTSV in healthy population among different provinces.

To explore the potential source of the high heterogeneity, we did some subgroup analysis by gender, age, occupation and province. The detail results of subgroup analysis were shown in **Table 2**. Stratification by gender, the pooled prevalence of male was 5.3% (95%CI: 3.3%-7.2%), and female was 5.2% (95%CI: 3.4%-7.0%). Stratification by age, the pooled seroprevalence of less than age of 40 years was 4.1% (95%CI: 2.4%-5.7%), and more than age of 40 years was 4.9% (95%CI: 2.9%-7.0%). Stratification by occupation, the pooled seroprevalence of farmer was 6.1% (95%CI: 3.4%-8.9%) and others (mainly are students) was 3.3% (95%CI: 2.4%-4.2%). Seropositive rates were comparable among sampling years, seroprevalence in the years before 2012 and after 2012 was 3.1% (1.9%-4.4%) and 5.2% (4.6%-5.9%), respectively. Stratification by testing method, seroprevalence in the method of I-ELISA and D-ELISA was 6.2% (5.4%~7.1%) and 1.8% (1.1%~2.5%), respectively. Seroprevalence of SFTSV in people who lived in the same village with the patient and lived in a different village was 6.5% (4.7~8.3) and 4.1% (3.1%~5.1%), respectively. We also found seroprevalence in endemic area was 4.3% (3.1%~5.6%) and in rural village was 4.3% (3.2%~5.4%). Additionally, the pooled seroprevalence was 5.9% (95%CI: 4.7%-7.0%) in Zhejiang province, 3.9% (95%CI: 1.3%-6.4%) in Shandong province, 4.9% (95%CI: 4.1–5.8%) in Anhui province, 6.9% (5.5%~8.2%) in Hubei province and 0.7% (95%CI: 0.2%-1.1%) in Jiangsu province. Forest plots for the meta-analysis in different provinces were shown in **Fig 4**.

**Table 2:** The seroprevalence of total antibodies (IgG/IgM) against SFTSV in different subgroups of humans in China.

Characters	No. studies	P ^a^ (%)	*95%CI* for P ^a^	Heterogeneity		Model
				*P* ^*b*^	*I*^*2*^ (%)	
Gender						
Male	11	5.3	3.3~7.2	<0.001	96.4	R
Female	11	5.2	3.4~7.0	<0.001	97.1	R
Age, years						
<40	9	4.1	2.4~5.7	<0.001	96.0	R
≥40	10	4.9	2.9~7.0	<0.001	97.6	R
Occupation						
Farmer	7	6.1	3.4~8.9	<0.001	96.7	R
Others*	6	3.3	2.4~4.2	0.301	17.9	F
Province						
Zhejiang	3	5.9	4.7~7.0	0.256	26.5	F
Shandong	6	3.9	1.3~6.4	0.001	95.5	R
Jiangsu	4	0.7	0.2~1.1	0.001	81.6	R
Anhui	3	4.9	4.1~5.8	0.147	47.9	F
Hubei	2	6.9	5.5~8.2	0.206	37.5	F
Quality						
High	5	4.7	2.2~7.1	<0.001	96.4	R
Moderate	15	3.8	2.4~5.2	<0.001	97.6	R
Area						
Endemic	14	4.2	2.8~5.5	<0.001	95.7	R
Population						
Rural	17	4.2	3.0~5.3	<0.001	96.3	R
Case villages	10	6.5	4.7~8.3	<0.001	89.5	R
Control villages	3	4.1	3.1~5.1	0.178	38.9	F
Sampling time						
≤2012	11	3.1	1.9~4.4	<0.001	96.8	R
>2012	7	5.2	4.6~5.9	0.257	22.6	F
Testing method						
I-ELISA	10	6.2	5.4~7.1	<0.001	73.3	R
D-ELISA	11	1.8	1.1~2.5	<0.001	88.2	R
Total	21	4.3	3.2~5.5	<0.001	97.3	R

*Abbreviations*: F, fixed model; R, random model.

P^a^. The seroprevalence of total antibodies (IgG/IgM) against SFTSV.

P^b^. P value of Q-test for heterogeneity test. When the P value was less than 0.10, the random effects model was used to assess the summary seroprevalence.

Others*: Students are the main portion.

### Publication bias and sensitive analysis

**Fig 5:**
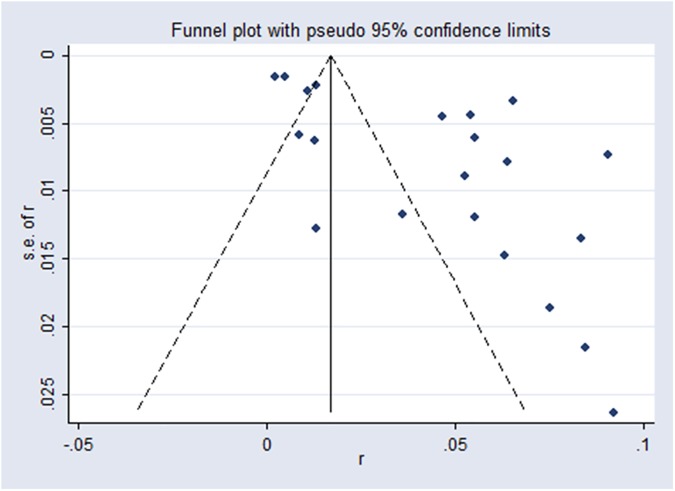
Funnel plot assessing publication bias in studies reporting SFTSV seroprevalence.

**Fig 6:**
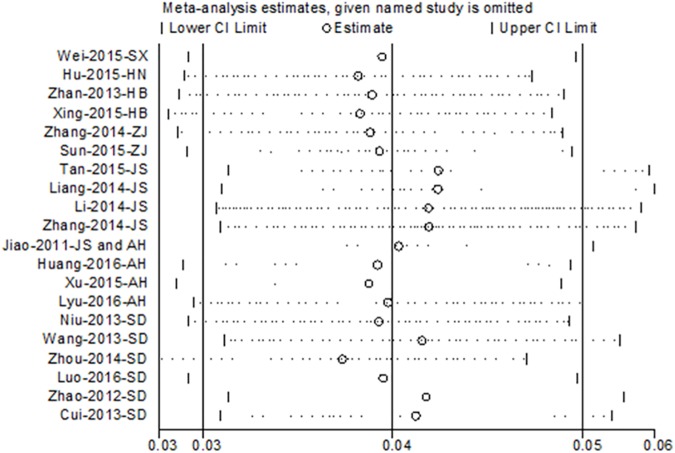
Sensitivity analysis of the summary seroprevalence of SFTSV. Results were computed by omitting each study in turn under random-effects model.

Potential publication bias of the included articles were evaluated by Begg’s funnel plots and Egger’s test. The shapes of the funnel plot was asymmetry evidently which gives rise to suspected publication bias (**Fig 5**). The Egger’s test results demonstrated evidence of publication bias, however, the Begg’s bias test was not significant for studies reporting seroprevalence of SFTSV (data not shown). A leave-one-out analysis which was performed to assess the impact of the individual study on the pooled estimates. Sensitive analysis showed that no single study qualitatively altered the pooled seroprevalence estimates, providing evidence of the stability of the meta-analysis (**Fig 6**).

## Discussion

SFTS as a global concerned infectious disease has posed a great threat on human health in East Asia. China is one of the most important endemic areas, more than 5000 cases of SFTS were reported in China from 2011 to 2014 [43].

This systematic review and meta-analysis was performed to evaluate the seroprevalence of SFTSV in Chinese population. Twenty one articles compromising a total of 23,848 individuals were included for analysis. Published seroprevalence ranged from 0.23% to 9.17%. We found that, among healthy population in China, the overall pooled seroprevalence of SFTSV was 4.3% (95%CI: 3.2%-5.4%). It was higher than a recent investigation in Korean population with a seroprevalence of 2.1% based on 1069 serum samples [44]. Sensitivity analysis by omitting one study at a time did not have substantial impact on the result, indicating the pooled result of seroprevalence was robust. Considerable heterogeneity was found among studies that can be at least partly explained by occupation, the geographical location and the anti-SFTSV antibodies assay employed.

Previous study reported gender ratio of SFTS patients in China was about 1:1.15 (male/female), but it varied among different provinces [45]. In this study, we found the seroprevalence of SFTSV in male was approximate with that in female. In addition, epidemiologic investigations showed that the majority of SFTS patients included retired or unemployed citizens and farmers. [46, 47]. Stratified by occupation in this meta-analysis, we found the seroprevalence of SFTSV in farmer was higher than other occupation (mainly are students), which indicates that farmers are the high risk population to SFTSV infections. It is believed that farmers take the main agriculture activities such as grass mowing, raising cattle, grazing in the bushes where ticks is highly intensive and active [22]. This high exposure experience could prominently increase the risk to SFTSV infection. Appropriate protective measures for farmers to repel ticks should be took when working outdoors.

Age composition of SFTS patients was widely, previous studies had demonstrated that SFTS patients ranged from 1 to 93 years old, but most of patients were aggregated in 40–79 years [41]. Generally, age was considered as a critical risk factor for morbidity and mortality of SFTS [41]. However, the results of seroprevalence in healthy people among different age groups were controversial. Some studies reported an increasing trend with age about the seroprevalence of SFTSV in healthy population [22, 23, 28, 31, 34], while other studies failed to find any significant difference among different age groups [30, 33, 41–43]. In this meta-analysis, the pooled data indicated the seroprevalence of SFTSV in people over 40 years old was close to people who less than 40 years old. This result indicated that all age groups were susceptible to SFTSV infection, but only aged people is inclined to get severe disease and to be hospitalized or even died of SFTSV infection.

The infections rates of healthy population varied in different provinces. It reflects that incidences of the latent infection of SFTS are different in endemic regions. In this study, we found SFTSV seroprevalence was high in Henan, Hubei, Zhejiang, Shanxi, Anhui and Shandong provinces, and was relatively low in Jiangsu provinces with seroprevalence of 0.7% (95%CI: 0.2~1.1). However, this result should be interpreted with cautious because of limiting number of study and sample size in some provinces, which may lead to lack of representativeness.

Additionally, different ELISA assay such as indirect ELISA (I-ELISA) and double-antigen sandwich ELISA system (D-ELISA) used to detect SFTSV-specific antibodies might also contribute to heterogeneity. Compared to the traditional I-ELISA, D-ELISA was considered having more higher sensitivity to detect total antibodies [33]. However, in the present meta-analysis, seroprevalence in the group of I-ELISA method was higher than that in the D-ELISA group. A potential explanation is that D-ELISA method was mainly used in Jiangsu province, where seroprevalence of SFTSV is relatively lower than other provinces.

Seroprevalence was also observed between different years, seroprevalence in the year after 2012 was higher than that before 2012, indicating ongoing and intensified circulation of SFTSV in endemic areas of China. Another discrepancy may be attributed to the sampling season, sampling method, previous contacting with animals (such as raising domestic animals, especially goats), or antibody types (total antibodies or IgG/ IgM antibody) detection in different studies.

The advantage of this systematic review and meta-analysis is that good quality studies from many centers were pooled for a relatively large sample size, but there are several limitations also should be addressed. Firstly, significant heterogeneity was detected across studies. Although we did some subgroup analysis to identify sources of heterogeneity, many unmeasured factors may have influenced the results. Secondly, funnel plots and Begg’s tests indicated that publication bias might be exist in the present study, which may distort the estimates of seroprevalence, so that the results should be interpreted with cautions.

In summary, data from 21 published studies suggested that SFTS is circulated widely in China and could be a cause of considerable health problems in the country. Surveillance efforts on mild or asymptomatic infections among endemic persons are needed. Further subtle-designed studies are still needed to describe the exact epidemiology of the disease at a national level in other parts of China.

## Supporting information

S1 Dataset Data for meta-analysis of overall and subgroups. (XLS)

S1 Table PRISMA 2009 Checklist. (DOC)

S1 Text PRISMA 2009 Checklist flow diagram. (DOC)
