# Supplementary material for: Seroprevalence of severe fever with thrombocytopenia syndrome virus in China: A systematic review and meta-analysis
Source: PLoS One. 2017 Apr 11;12(4):e0175592. doi: 10.1371/journal.pone.0175592 (PMC5388504; doi:10.1371/journal.pone.0175592)
Supplement: S1 Text — (DOC) [file pone.0175592.s003.doc]

**Screening**

**Included**

**Eligibility**

**Identification**

Records identified through database searching
(n = 599 )

Additional records identified through other sources
(n = 70 )

Records after duplicates removed
(n = 523 )

Records screened
(n = 523 )

Records excluded
(n = 471 )

Full-text articles assessed for eligibility
(n = 52 )

Full-text articles excluded, with reasons
(n = 31 )

Studies included in qualitative synthesis
(n = 21 )

Studies included in quantitative synthesis (meta-analysis)
(n = 21 )
